# Supplementary material for: Inspiring diverse researchers in Virginia: Cultivating research excellence through a career-building program
Source: J Clin Transl Sci. 2024 Jan 22;8(1):e27. doi: 10.1017/cts.2024.12 (PMC10880007; doi:10.1017/cts.2024.12)
Supplement: Mata-McMurry et al. supplementary material [file S2059866124000128sup001.pdf]

iDRIV Application Submission Survey

Thank you for your interest in the iDRIV Scholars Program! For complete information about the iDRIV program including eligibility please visit our website: https://www.ithriv.org/idriv-program

Please complete and submit this application and be sure to include a CV or NIH biosketch. For questions please reach to Jennifer Phillips at jvp8a@virginia.edu.

Applicant Information

**First Name**	
**Last Name**	
**Email Address**	
**Degree**	
**Department/ Institute**	
**Division (if applicable)**	

Current Position

* must provide value

- ○ Associate Professor, less than 5 years
- ○ Assistant Professor
- ○ Fellow
- ○ Post Doctoral Fellow
- ○ Other (please specify)

Preferred pronouns

- ◯ She
- ◯ He
- ◯ They
- ◯ Ze
- ◯ Prefer to self-identify

Do you meet the NIH definition of underrepresented persons in biomedical research?

https://diversity.nih.gov/about-us/population-underrepresented

* must provide value

- ◯ Yes
- ◯ No
- ◯ Not sure

The following questions are optional. Demographic data will be combined and used only to fulfill our required programmatic reporting to NIH.

What best describes your gender identification? (Please select all that apply)

* must provide value

- ◯ Cis-woman
- ◯ Trans woman
- ◯ Cis-man
- ◯ Trans-man
- ◯ Non-Binary
- ◯ Prefer not to disclose
- ◯ Another gender identity not listed

Which categories describe you? (Please select all that apply)

* must provide value

- ☐ African, African-American, or Black: A person having origins in any of the black racial groups of Africa or the Caribbean
- ☐ American Indian or Native Alaskan: A person having origins in any of the original peoples of North and South America (including Central America). For example, Blackfeet tribe, Cherokee Nation.
- ☐ Arab American, Middle Eastern, North African
- ☐ East Asian or Asian-American: A person having origins in any of the original peoples of East Asia including, for example, China, Japan, Mongolia, North Korea, South Korea, and Taiwan

- ☐ South Asian or Asian American: a person having origins in any of the original peoples of South Asia including, for example, Indian, Afghanistan, Iran, Nepal, Pakistan, Sri Lanka,
- ☐ and Bangladesh
- ☐ Hispanic or Latina/o: A person of Cuban, Mexican, Puerto Rican, or South or Central American, or other culture or origin
- ☐ Native Hawaiian or Pacific Islander: A person having origins in any of the original peoples of Hawaii, Guam, Samoa, or other Pacific Islands
- ☐ White: A person having origins in any of the original peoples of Europe
- ☐ Multi racial: A person consisting of 2+ ethnicities
- ☐ Prefer not to disclose
- ☐ Prefer to self-identify

What best describes your sexual orientation? (Please select all that apply)

* must provide value

- ☐ Straight/Heterosexual
- ☐ Lesbian
- ☐ Gay
- ☐ Queer
- ☐ Asexual
- ☐ Unsure
- ☐ Prefer not to disclose
- ☐ Another sexual orientation not listed

With which ethnicity do you most identify? (Please select all that apply)

* must provide value

- ◯ Hispanic/Latino/Latina/Latinx/Spanish
- ◯ Non-Hispanic/Latino/Latina/Latinx/Spanish
- ◯ Prefer not to disclose

Do you have a disability (defined as a physical or mental impairment that substantially limits one or more major life activities)?

The NIH defines an individual with a disability as a person with physical or mental impairment that substantially limits one or more major life activities. More info can be found here: https://extramural-diversity.nih.gov/diversity-matters/individuals-with- disabilities

* must provide value

- ◯ Yes
- ◯ No
- ◯ Prefer not to disclose

Are you from a disadvantaged background?
The NIH definition of disadvantaged background can be found here: https://extramural-diversity.nih.gov/diversity-matters/disadvantaged-backgrounds

* must provide value

- ◯ Yes
- ◯ No
- ◯ Prefer not to disclose

Please indicate your veteran status (Please select all that apply)
More info on veteran categories can be found here: https://www.dol.gov/agencies/ofccp/vevraa/self-id-form

* must provide value

- ◯ I am not a veteran of the U.S military (I did not serve in the military)
- ◯ I am a disabled veteran of the U.S military
- ◯ I am a recently separated veteran of the U.S military
- ◯ I am an active wartime or campaign badge veteran
- ◯ I am an armed forces service medal veteran
- ◯ I am NOT a protected veteran. (I served in the military but do not fall into any veteran categories listed above.)
- ◯ Prefer not to disclose

Are you a U.S. citizen/permanent resident?

* must provide value

- ◯ Yes
- ◯ No
- ◯ Prefer not to disclose

Scientific Mentor Information

First Name of Scientific Mentor (if applicable)

Last Name of Scientific Mentor (if applicable)

Scientific Mentor's Email Address

How often are you meeting with your research mentor?

- ◯ Weekly
- ◯ Every other week
- ◯ Monthly
- ◯ Occasionally (once every few months)
- ◯ Never
- ◯ I don't have a research mentor

Applicants: Please answer the following questions below as completely as possible.

Provide a brief description of the type of research you are doing or wish to do?

* must provide value

What are your short term research goals (this coming year)?

For example: Grant applications (focusing on career development awards, federal or foundation funding)? Presentation at a National Meeting? Publications? etc.

* must provide value

What are your long term research goals (5 year plan)?

For example: Grant application (focusing on career development awards, federal or foundation funding)? Presentation at a National Meeting? Publications? Career Advancement? Etc.

* must provide value

How much protected research time do you currently have?

What is your current promotion and tenure track? (if applicable)

(https://faculty.med.virginia.edu/facultyaffairs/advancement/pandt/faculty-tracks/)

What are the biggest challenges you are facing that may impact your research?

* must provide value

Describe an experience related to your underrepresented status that has impacted you as a researcher or has impacted your research.

What do you hope to gain from participation in this program?

* must provide value

Please upload a copy of your current CV or NIH Biosketch

* must provide value

Submit

Powered by REDCap
